# Effects of clonal integration on the invasive clonal plant *Alternanthera philoxeroides* under heterogeneous and homogeneous water availability

**DOI:** 10.1038/srep29767

**Published:** 2016-07-15

**Authors:** Wen-Hua You, Cui-Min Han, Chun-Hua Liu, Dan Yu

**Affiliations:** 1 The National Field Station of Lake Ecosystem of Liangzi Lake, College of Life Science, Wuhan University, Wuhan, 430072, P.R. China; 2 Institute of Environment and Ecology, College of the Environment and Safety Engineering, Jiangsu University, Zhenjiang, 212013, P.R. China

## Abstract

Many notorious invasive plants are clonal, living in heterogeneous or homogeneous habitats. To understand how clonal integration affects the performance of these plants in different habitat conditions, an 8-week greenhouse experiment was conducted: ramet pairs of *A. philoxeroides* were grown in two habitats, either heterogeneous or homogeneous in water availability, with the stolon connections either severed or kept intact. Under heterogeneous water availability, compared with ramets in homogeneous habitats, clonal integration significantly promoted the growth and photosynthetic performance of water-stressed apical ramets, whereas it only increased the photosynthetic performance but did not affect the growth of water-stressed basal ramets. Moreover, clonal integration markedly increased the root/shoot ratios of ramets grown in habitats with high water supply but decreased it under low water availability. Under homogeneous water availability, stolon connection (clonal integration) did not influence the growth, photosynthetic performance and biomass allocation of water-stressed ramets, but it significantly promoted the growth of well-watered ramets in both apical and basal sections. These findings deepen our understanding of the bidirectional and differentiated (mainly acropetal) clonal integration of *A. philoxeroides*, suggesting that the invasive plant *A. philoxeroides* can benefit from clonal integration in both heterogeneous and homogeneous habitats.

Plant invasion poses a great threat to biodiversity, environment and economy both globally and locally^1^,^2^. An important factor in invasion success is the characteristics of the plant species, such as clonal traits^3^. Many of the most notorious alien invasive plants have the capacity for vigorous clonal propagation^3^,^4^,^5^,^6^. Recently, some studies have demonstrated that the invasiveness of alien clonal plants may be closely related to clonal traits such as clonal integration (i.e., the reciprocal translocation of resources between interconnected ramets)^7^,^8^,^9^,^10^,^11^. For example, the invasive clonal plant *Alternanthera philoxeroides* (alligator weed) can form dense stands through clonal propagation that expel almost all the other species in aquatic ecosystems^12^. Clonal integration has been considered to be an important factor for the growth, spread and invasion of this invasive species in different habitat conditions^4^,^5^,^11^,^13^,^14^.

Natural habitats exhibit both heterogeneous and homogeneous distribution of essential resources^15^,^16^. Clonal propagation results in extensive structures that occupy large areas and are almost bound to experience both heterogeneous and homogeneous habitats^10^,^16^. Numerous studies have addressed the role of clonal integration for clonal plants in coping with heterogeneous resources, such as nutrients, light, water, space and others^4^,^5^,^7^,^15^,^17^. These studies showed that clonal integration can facilitate the colonization and growth of ramets under stressful conditions^7^,^17^,^18^, improve the tolerance of individual ramets to physical disturbances^19^,^20^,^21^ and help genets to survive and recover after severe environmental changes^22^,^23^. However, most of these studies have focused on the positive effects of clonal integration on the apical (daughter or offspring) ramets exposed to stress conditions or low-resource environments, whereas few studies have investigated the outcome of clonal integration effects when basal (mother or parent) ramets were exposed to stressful habitats while apical ramets were in resource-rich habitats^13^,^24^. Moreover, it is believed that clonal integration may have little effect on the performance of clonal plants when resource availability is homogeneously distributed^10^,^16^,^25^, although it may affect the performance of clonal plants when they contain ramets that are in different stages of development and differ in their ability to take up resources^16^. In a recent study, using a conceptual model, Dong *et al*.^16^ found that clonal integration may also have a positive effect on the performance of clonal plants when connected ramets differ in uptake ability in homogeneous high-resource environments. However, how clonal integration affects the performance of clonal plants in homogeneous habitats is still not well understood.

Physiological variables such as photosynthetic performance (photochemical efficiencies and photosynthetic rates) are key properties in assessing plant fitness and general performance^4^,^15^,^17^. Therefore, the knowledge of clonal plants’ physiological responses to environmental factors, mediated by clonal integration, will provide insights into functional mechanisms^4^,^15^,^21^. Moreover, compared with non-clonal plants, the ramets of clonal plants in favourable patches can have a proportionally larger biomass allocation to organs (roots or leaves) that are associated with resource uptake, resulting in a specialization of ramets to acquire a locally abundant resource instead of a scarce one^26^. This specialization may improve the exploitation of resources and overall performance throughout the entire clonal system^7^,^26^. Biomass allocation and physiological variables can both contribute to the performance of clonal plants^7^,^11^; however, the understanding of the responses of invasive clonal plants to clonal integration in different habitat conditions (especially in homogeneous habitats) remains limited.

To understand how clonal integration affects the performance of the invasive plants in both heterogeneous and homogeneous habitats, a greenhouse experiment was conducted to investigate the effects of clonal integration on the growth, photosynthetic performance and biomass allocation of *A. philoxeroides* under heterogeneous and homogeneous water availability. Ramet pairs of *A. philoxeroides* were grown in two habitats, either heterogeneous (well watered or water stressed) or homogeneous in water availability, with stolon connections either severed or kept intact. Specifically, we tested the following hypotheses. (1) In heterogeneous habitats, clonal integration will increase the photosynthetic performance and growth of water-stressed (recipient) ramets, whereas it will decrease or have little effect on the photosynthetic performance and growth of well-watered (donor) ramets of *A. philoxeroides* in both the apical and basal parts. (2) Clonal integration will influence the biomass allocation of *A. philoxeroides* in heterogeneous habitats but will not affect the biomass allocation in homogeneous habitats. According to the theory of labour division^26^, in heterogeneous habitats, we predict that clonal integration will increase biomass allocation to roots for the well-watered ramets (where a belowground resource such as water is comparatively more abundant), whereas it will increase biomass allocation to shoots for the water-stressed ramets (where an aboveground resource such as light or space is comparatively more abundant). (3) Based on the conceptual model proposed by Dong *et al*.^16^, we predict that stolon connection (clonal integration) will increase the growth of ramets of *A. philoxeroides* in homogeneous habitats.

## Material and Methods

### Ethics Statement

Plant material used in this experiment was collected from natural plant populations at the National Field Station of Freshwater Ecosystem of Liangzi Lake (N 30°05′–30°18′, E 114°21′–114°39′).The plant species was common and naturally distributed in this area. No specific permissions were required for these locations. This study did not involve any endangered or protected species.

### Plant species

*Alternanthera philoxeroides* (Mart.) Griseb. (Amaranthaceae), or alligator weed, originating from South America, is a clonal weed that causes serious economic and environmental problems worldwide^12^,^27^. It is stoloniferous and amphibious, growing in both riparian and terrestrial habitats^12^. This species is one of the world’s worst invasive weeds and listed as one of the 16 worst alien invasive weeds in China^27^,^28^. *A. philoxeroides* often suffers natural disturbances, such as herbivory, mowing and trampling, which may fragment its clones in to pieces^21^,^29^,^30^,^31^. In China, *A. philoxeroides* has extremely low genetic diversity^32^,^33^, and clonal integration plays an important role in determining its growth and spread^4^,^7^,^13^,^14^,^21^.

### Experimental design

**Figure 1:**
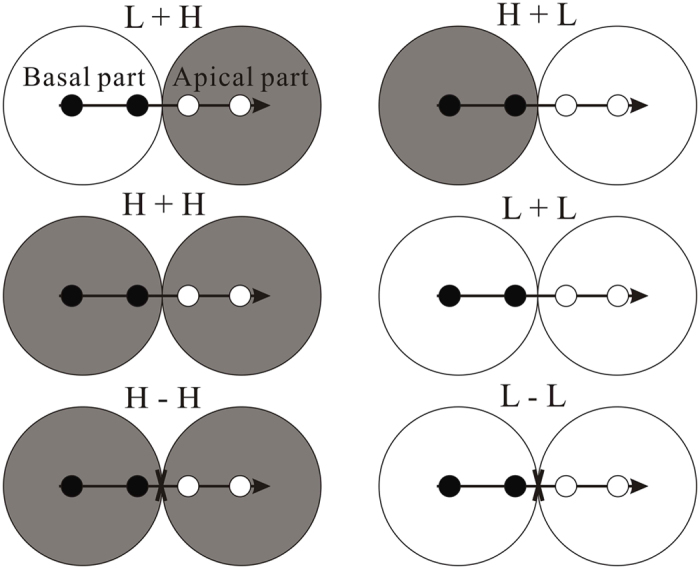
Schematic representation of the experimental design. There were six experimental treatments involving clonal integration and water availability. Clonal fragments of the invasive plant *A. philoxeroides*, each consisting of two basal ramets (black circles) and two apical ramets (white circles) with a stolon apex (horizontal arrow), were grown either in the well watered habitats (grey) or in the water stressed habitats (white), and with the stolon connections between basal and apical ramets were either intact or severed (fork). See text for additional explanation.

This experiment was conducted in a greenhouse under natural sunlight (1200–1400 μmol m^−2^ s^−1^) and at ambient temperature (20–28 °C) at The National Field Station of the Lake Ecosystem of Liangzi Lake, Wuhan University (N 30°05′–30°18′, E 114°21′–114°39′). In mid-April 2012, the source material of *Alternanthera philoxeroides* was collected from Liangzi Lake in Hubei province of China and then propagated in the greenhouse. The plants used in this experiment were 48 similar-sized clonal fragments of *A. philoxeroides* (tip cuttings, 12.35 ± 0.15 cm in length, 0.42 ± 0.09 g in dry mass; means ± SE), each consisting of a stolon with four ramets and a stolon apex. Each clonal fragment was divided into two parts, one termed the ‘basal part’, consisting of two relatively old ramets (close to the mother ramets), and the other the ‘apical part’, consisting of two relatively young ramets (distal to the mother ramets) and a stolon apex^11^. Each clonal fragment was randomly assigned such that the two ramets of the basal part were placed within the basal pot, and the other two ramets and the apex of the apical part were placed within the apical pot (Fig. 1).

Six experimental treatments involving clonal integration and water availability were performed in this study: (1) heterogeneous water supply (low in the basal part and high in the apical part) with the stolon connected, L+H; (2) heterogeneous water supply (high in the basal part and low in the apical part) with the stolon connected, H+L; (3) homogeneous high water supply with the stolon connected, H+H; (4) homogeneous low water supply with the stolon connected, L+L; (5) homogeneous high water supply with the stolon severed, H−H; (6) homogeneous low water supply with the stolon severed, L−L (Fig. 1). Treatments 1–4 were used to test the effects of clonal integration under heterogeneous water availability using a homogeneous–heterogeneous approach, whereas Treatments 3–6 were used to examine the effects of clonal integration under homogeneous water availability using a stolon severing approach (Fig. 1)^10^. Each treatment was replicated eight times.

There were two levels of water availability. Under high water availability, the experimental pots (20 cm in diameter, 15 cm tall) were supplied with sufficient lake water (TN 0.6 mg L^−1^, TP 0.05 mg L^−1^, pH 7.8) to keep the soil saturated with water (well watered). In the low water availability treatment, low amounts of water (100 mL lake water per pot every other day) were applied only to maintain plant growth without wilting (water stressed). All the pots were filled with a mixture of washed sand and lake mud (TN 2.94 mg g^−1^, TP 0.15 mg g^−1^) at a volume ratio of 1:1. During the experimental period, all the apical and basal ramets survived. To avoid the effects of possible environmental patchiness within the greenhouse, all the experimental units (basal pot + apical pot) were randomly arranged at the beginning of the experiment and systematically repositioned in the greenhouse every other week so that each experimental unit experienced all possible conditions. The experiment was conducted for eight weeks, and the plants were harvested on July 6th, 2012.

### Measurements

Three days before harvesting the plants, the minimum (*F*_0_) and the maximum (*F*_m_) fluorescence yield were measured after dark adaptation for at least 20 minutes using leaf clips. Measurements were conducted on a fully developed, healthy leaf of the second-youngest ramet of both the apical and basal part using a portable chlorophyll fluorometer (Diving-PAM, Walz, Effeltrich, Germany) with the saturation pulse method. The maximum quantum yield of PSII (*F*_v_/*F*_m_) was calculated as (*F*_m_−*F*_0_)/*F*_m_^34^, which is a sensitive indicator of plant photosynthetic performance that usually decreases significantly under environmental stress^5^,^7^. Leaf gas exchange was also measured using a Li-6400 portable photosynthesis system (Li-Cor Biosciences, Lincoln, NE, USA). The leaves used for gas exchange measurements were the ones opposite to the leaves used for determination of *F*_v_/*F*_m_. The net photosynthetic rate (*P*n) was measured at a CO_2_ concentration of 400 μmol mol^−1^ (near the ambient CO_2_ concentration)^4^ and a photo flux density of 1200 μmol m^−2^ s^−1^ in area-based units (the light saturation point of leaves of *A. philoxeroides* obtained from a preliminary experiment).

At the final harvest, the number of ramets and the total stolon length of the apical and basal ramets were measured separately. Then, the apical and basal parts of the *A. philoxeroides* plants were harvested and separated into leaves, stolons and roots, and their biomass was determined after drying at 70 °C for 72 h.

### Statistical analysis

All data were analysed using fixed-model one-way analysis of variance (ANOVA) after correction for non-normality and heteroscedasticity by logarithmic transformation or, in the case of proportions, by angular transformations. The growth measurements (biomass, ramet number and stolon length) of the apical part, the basal part and the whole clonal fragment were separately compared between experimental treatments. The physiological measurements (*F*_v_/*F*_m_ and *P*n) and shoot/root ratios of the apical part and the basal part were also separately compared between experimental treatments. Post-hoc pair-wise comparisons of the means were performed to examine differences between the treatments using the Studentized Tukey’s HSD for multiple comparisons. Statistical significance was assigned at *P* < 0.05. All data analyses were performed using SPSS 17.0 (SPSS, Chicago, IL, USA).

## Results

### Growth

**Figure 2:**
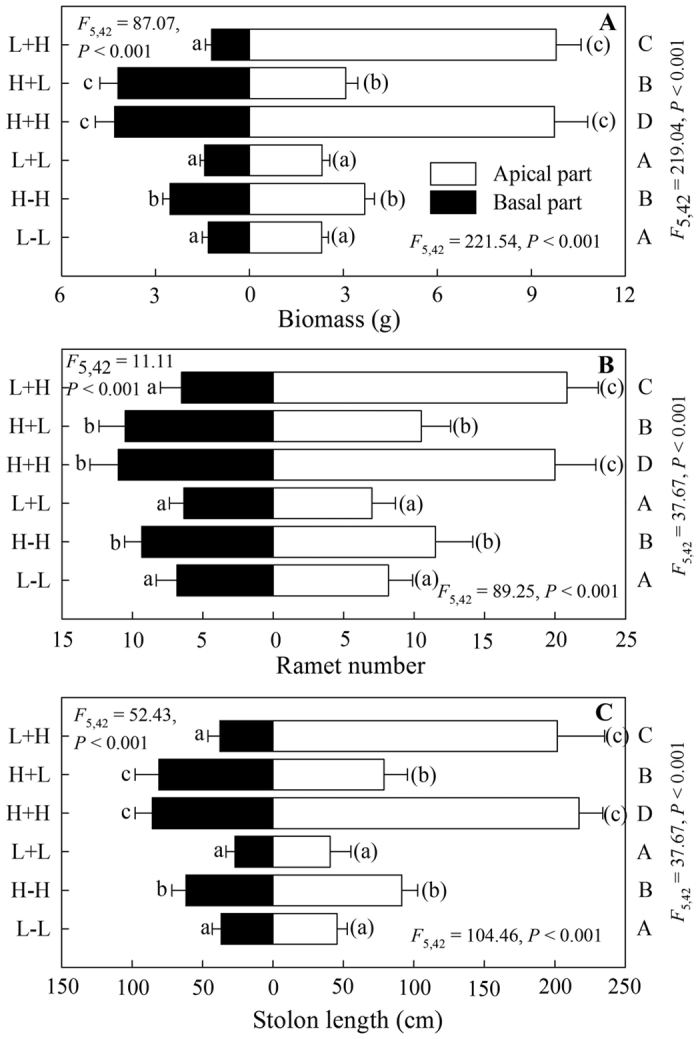
Effects of experimental treatments on total biomass (**A**), ramet number (**B**) and stolon length (**C**) of the invasive plant *A. philoxeroides* in the apical parts, basal parts and the whole clonal fragments. Data indicate the means ± SE. Bars sharing the same letter are not significantly different at *P* = 0.05 (one-way ANOVA with Studentized Tukey’s HSD test).

In the heterogeneous treatments, for the apical parts, the growth measures (biomass, ramet number and stolon length) of ramets with high water supply were much higher than for ramets with low water supply (Fig. 2). Compared to their corresponding ramets with homogeneous water supply (L+L), the growth of water-stressed ramets under heterogeneous water availability (H+L) was significantly better (Fig. 2). However, there was no significant difference between the growth of apical ramets in the two well-watered (H+H and L+H) treatments (Fig. 2). For the basal parts, well-watered ramets grew better than water-stressed ramets (Fig. 2). There were no significant differences between the growth of basal ramets under the same water availability (Fig. 2). For the whole plants, the growth of H+H was best, followed by L+H, H+L and L+L (Fig. 2).

In the homogeneous treatments, stolon connection (clonal integration) had no significant effect on the growth of apical ramets, basal ramets and the whole plants under low water availability (Fig. 2). However, with a high water supply, stolon connection greatly increased the growth of apical ramets, basal ramets and the whole clonal fragments (Fig. 2).

### Photosynthetic performance

**Figure 3:**
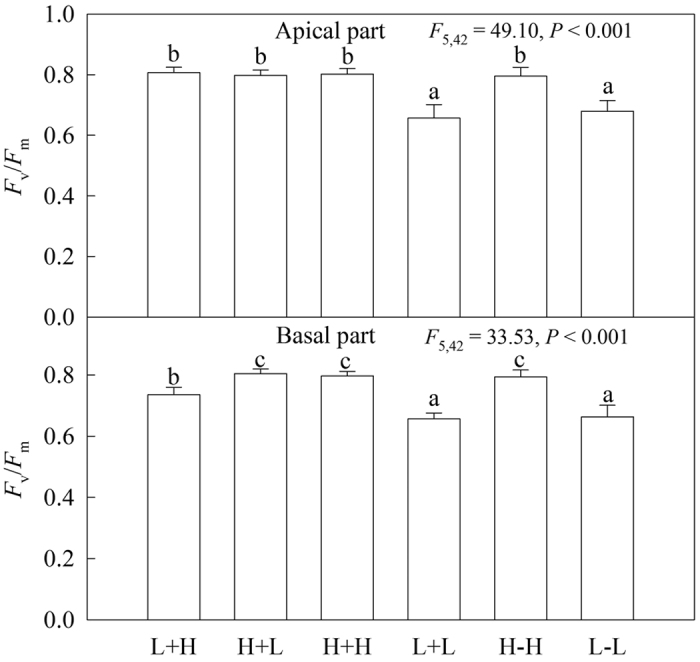
Effects of experimental treatments on the maximum quantum yield of photosystem II (*F*_v_/*F*_m_) of the invasive plant *A. philoxeroides* in the apical parts (**A**) and basal parts (**B**). Data indicate the means ± SE. Bars sharing the same letter are not significantly different at *P* = 0.05 (one-way ANOVA with Studentized Tukey’s HSD test).

**Figure 4:**
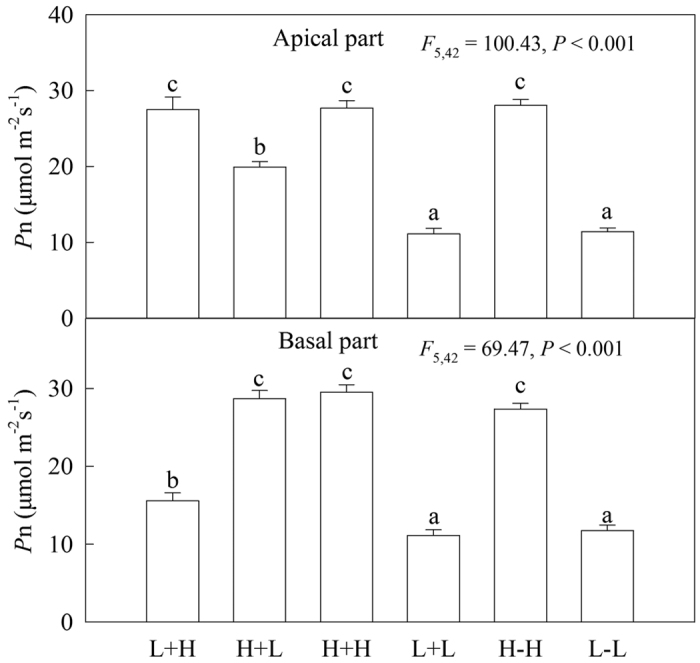
Effects of experimental treatments on the net photosynthetic rates (*P*n) of the invasive plant *A. philoxeroides* in the apical parts (**A**) and basal parts (**B**). Data indicate the means ± SE. Bars sharing the same letter are not significantly different at *P* = 0.05 (one-way ANOVA with Studentized Tukey’s HSD test).

In the heterogeneous treatments, the *F*_v_/*F*_m_ values of water-stressed apical ramets in the L+L treatment were significantly lower than for apical ramets in other treatments, whereas these values in other treatments (L+H, H+L and H+H) showed no significant differences (Fig. 3A). The net photosynthetic rate (*P*n) of apical ramets in the L+L treatment was significantly lower than for apical ramets in the H+L treatment, whereas these values were significantly higher in the treatments of L+H and H+H than in the H+L treatment (Fig. 4A). For the basal ramets, the *F*_v_/*F*_m_ values of water-stressed ramets under heterogeneous water availability (L+H) were significantly higher compared to their corresponding ramets with homogeneous water supply (L+L), while the values in the L+H treatment were lower than in the other two treatments (Fig. 3B). Moreover, the effects of experimental treatments on the net photosynthetic rates of ramets were similar to the effects on *F*_v_/*F*_m_ (Fig. 4B).

In the homogeneous treatments, for both the apical and basal ramets, the photosynthetic performance (*F*_v_/*F*_m_ and *P*n) of the well-watered ramets was significantly higher than for the water-stressed ramets (Figs 3 and 4). Stolon connection had no significant effects on the photosynthetic performance (*F*_v_/*F*_m_ and *P*n) of either apical or basal ramets under homogeneous water availability (Figs 3 and 4).

### Root/shoot ratio

**Figure 5:**
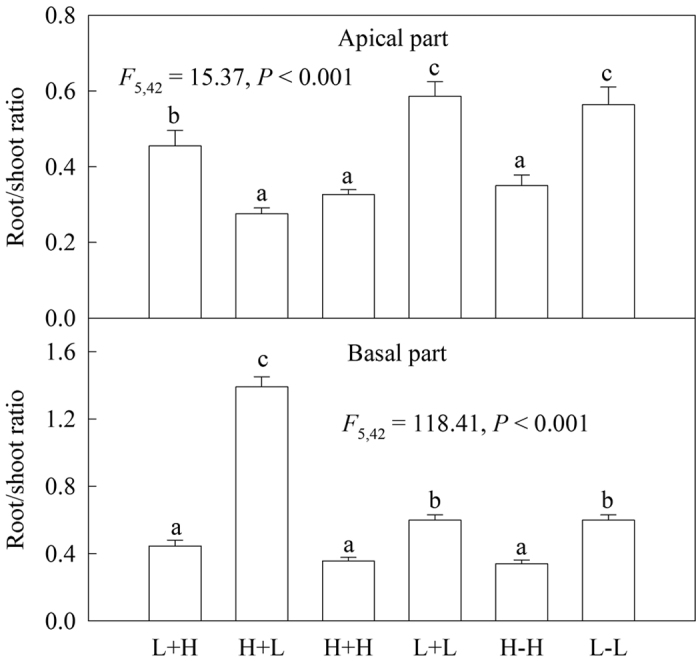
Effects of experimental treatments on the root/shoot ratios of the invasive plant *A. philoxeroides* in the apical parts (**A**) and basal parts (**B**). Data indicate the means ± SE. Bars sharing the same letter are not significantly different at *P* = 0.05 (one-way ANOVA with Studentized Tukey’s HSD test).

In the heterogeneous treatments, the root/shoot ratios of both apical and basal ramets with homogeneous high water supply (H+H) were significantly lower than with homogeneous low water supply (L+L) (Fig. 5). Interestingly, for both apical and basal ramets, the root/shoot ratios of well watered ramets under heterogeneous water availability were much higher than for the corresponding ramets with a homogeneous water supply, whereas these values were significantly lower for water-stressed ramets under heterogeneous water availability than for the corresponding ramets with a homogeneous water supply (Fig. 5). However, in the homogeneous treatments, the root/shoot ratios of both apical and basal ramets with high water supply (H+H and H−H) were significantly higher than for the ramets with low water supply (L+L and L−L) (Fig. 5). However, stolon connection did not significantly affect the root/shoot ratios of either apical or basal ramets under homogeneous water availability (Fig. 5).

## Discussion

### Effects of clonal integration on *A. philoxeroides* in heterogeneous habitats

As hypothesized, in heterogeneous habitats, clonal integration significantly increased the photosynthetic performance (*F*_v_/*F*_m_ and *P*n) and growth (total biomass, number of ramets and total stolon length) of water-stressed ramets of *A. philoxeroides* in the apical parts. These results were most likely because the relatively older ramets (well watered) in the basal parts supported the growth of the interconnected young apical ramets, facilitating the enhancement of photosynthetic performance and the production of new tissue due to the acropetal (from basal ramets to apical ramets) translocation of carbohydrates and nutrients^5^,^7^,^17^. This finding agrees with the results obtained in previous studies on several invasive clonal plants including *A. philoxeroides*^7^,^11^, *Myriophyllum aquaticum*^5^ and *Eichhornia crassipes*^35^, which showed that clonal integration can improve the photosynthetic performance, growth and clonal propagation of daughter ramets, thus helping genets to occupy open space under stress conditions or in low-resource environments.

When the basal ramets were water stressed, the interconnected well-watered ramets in the apical parts only increased the photosynthetic performance of the basal ramets but did not affect their growth. These findings demonstrated that the benefits of clonal integration for the basal ramets in terms of photochemical activity and photosynthesis were mainly due to the increase in carbohydrates and nutrients (such as nitrogen) supported by well watered ramets in the apical parts^15^,^17^. However, the benefit of clonal integration in photosynthetic performance was not translated into benefits in growth. This result may be explained by the following mechanisms. (1) Considering that growth is the result of the balance between photosynthesis and respiration, we believe that clonal integration may also increase respiration at a rate that counteract the benefits of an increased photosynthetic rate^15^. (2) The increase in carbohydrates resulting from the enhanced photosynthetic performance may be acropetally transported to the well-watered ramets in the apical parts to support the occupancy of space and spreading (habitat selection)^17^,^36^. Therefore, the water-stressed ramets in the basal parts benefited less from clonal integration than water-stressed ramets in the apical parts. These findings suggest that the clonal integration of *A. philoxeroides* in heterogeneous habitats is bidirectional^37^ and differentiated (more acropetal and less basipetal), promoting its expansion to favourable habitats^38^,^39^.

As we predicted, clonal integration significantly influenced the root/shoot ratios of *A. philoxeroides* in heterogeneous habitats, greatly increasing the root/shoot ratios of the well-watered ramets but decreasing the root/shoot ratios of the water-stressed ramets for both the apical and basal parts (Fig. 5). This result is probably because the belowground resource (i.e., water) was comparatively more abundant for the well-watered ramets, whereas the aboveground resource (light or space) was comparatively more abundant in water-stressed habitats. This result is consistent with previous findings for some other invasive clonal plants^5^,^7^,^9^, which demonstrated that the ramets of invasive clonal plants in favourable habitats can have a proportionally larger biomass allocation to organs associated with resource uptake, resulting in a specialization of ramets to acquire a locally abundant resource, i.e., ‘division of labour’^9^,^26^. Therefore, the effects of clonal integration on the biomass allocation pattern may improve the uptake of resources for *A. philoxeroides* and enhance its invasiveness in heterogeneous environments^11^.

### Effects of clonal integration on *A. philoxeroides* in homogeneous habitats

It is believed that clonal integration may have little effect on the performance of clonal plants when resource availability is homogeneously distributed^10^,^16^,^25^. Nevertheless, in this study, stolon connection (clonal integration) played a different role in homogeneous habitats. With a low water supply, clonal integration did not influence the photosynthetic performance, growth and biomass allocation of ramets in both the apical and basal sections. This result most likely occurred because low water availability was the main limiting factor for plant growth and survival, and there were no additional resources for the interconnected ramets to share. Therefore, clonal integration was less likely to occur under such stressful conditions.

In contrast, in habitats with high water availability, clonal integration significantly promoted the growth of ramets in both sections and the performance of the whole clonal fragments. This result is probably for the following reasons: (1) severing stolons resulted in mechanical damage and physiological stress, which may make the plants more vulnerable to pathogen infections^10^ (personal observation) and therefore decrease ramet performance for both the apical and basal parts in well watered habitats; and (2) given that the clonal fragments used in this study may contain ramets that are in different stages of development (i.e., a mother-daughter ramet system in which apical ramets are relatively younger and basal ramets are relatively older) and/or differ in the ability to take up resources, clonal integration between the apical and basal ramets in resource-rich habitats may still occur, probably due to the acropetal translocation of resources^16^, which may increase the general performance of the plants in homogeneous habitats with high water supply. Consequently, these findings suggest that clonal integration may be more important for the growth, spread and invasion of *A. philoxeroides* in resource-rich homogeneous habitats than in stressful homogeneous habitats. Moreover, our study may add support to the argument that clonal integration can contribute to the dominance of this invasive plant in habitats that appear to have relatively little fine-scale spatial patchiness (such as aquatic ecosystems)^16^.

## Conclusions

Through a greenhouse experiment, we show that the clonal integration of *A. philoxeroides* is bidirectional but differentiated (mainly acropetal) in heterogeneous habitats, and clonal integration may be more important for the growth, spread and invasion of *A. philoxeroides* in resource-rich homogeneous habitats than in stressful homogeneous habitats. These findings support our hypothesis that the invasive plant *A. philoxeroides* can benefit from clonal integration in both heterogeneous and homogeneous habitats, suggesting that the invasiveness of this plant may be closely related to clonal integration in diverse habitat conditions^5^,^7^,^13^,^14^,^40^. In this study, a mother-daughter ramet system (apical ramets are younger and basal ramets are older) was used to test clonal integration. This approach may be part of why clonal integration also had an impact in homogeneous environments and why acropetal translocation was dominant. Moreover, considering that the effect of clonal integration could depend on the resource type (i.e., water, nutrients or carbohydrates) being translocated^10^,^41^, further studies that involve other resources are required to explore the effects of clonal integration on the performance of invasive clonal plants to fully understand the roles of clonal integration in shaping such plants’ invasion success.

## Additional Information

**How to cite this article**: You, W.-H. *et al*. Effects of clonal integration on the invasive clonal plant *Alternanthera philoxeroides* under heterogeneous and homogeneous water availability. *Sci. Rep.* **6**, 29767; doi: 10.1038/srep29767 (2016).
